# Nationwide epinephrine auto-injector dispensing in Greece: a drug-utilization study providing insights into anaphylaxis epidemiology and emergency-treatment preparedness

**DOI:** 10.3389/falgy.2026.1906574

**Published:** 2026-09-14

**Authors:** George N. Konstantinou

**Affiliations:** 1 Department of Allergy and Clinical Immunology, 424 General Military Training Hospital, Thessaloniki, Greece; 2 Elliot and Roslyn Jaffe Food Allergy Institute, Division of Pediatric Allergy and Immunology, Department of Pediatrics, Icahn School of Medicine at Mount Sinai, New York, NY, United States

**Keywords:** Adrenaline auto-injector, anaphylaxis, drug utilization, epidemiology, epinephrine auto-injector, medication access, preparedness

## Abstract

**Background:**

Anaphylaxis is a serious systemic hypersensitivity reaction requiring prompt recognition and treatment, yet diagnosis-linked national surveillance is unavailable in many countries. Epinephrine auto-injector (EAI) dispensing cannot identify confirmed events or individual patients, but it is an epidemiologically relevant medication-use endpoint because EAIs are supplied to people with prior anaphylaxis or a clinically judged risk of future anaphylaxis.

**Objective:**

To quantify nationwide EAI dispensing in Greece as a drug-utilization and emergency-treatment-preparedness benchmark and, secondarily, derive scenario-based incidence-compatible and prevalence-equivalent indicators relevant to the epidemiology of anaphylaxis.

**Methods:**

This nationwide retrospective drug-utilization study analyzed aggregated net Anapen® DUO pack counts from reimbursed and non-reimbursed retail and hospital pharmacy channels during March 2015-March 2017, when it was the only EAI marketed nationally. Directly observed pack flow was primary, utilization by EAI strength was analyzed at the population level using age-equivalent denominators, expiry timing was examined conditionally, and scenario outputs were deterministic sensitivity analyses.

**Results:**

During 25 months, 33,986 twin-packs were recorded (153.2 packs per 100,000 person-years). The final 12-month rate was 154.0 per 100,000 population. Using age-equivalent population denominators, 150-microgram dispensing intensity was 5.8-fold higher than 300-microgram intensity. Reference scenarios yielded an incidence-compatible anaphylaxis indicator of 47.7–125.3 episodes per 100,000 person-years and prevalence-equivalent active EAI coverage of 51.7–133.0 persons per 100,000. Deliberately broader assumption ranges widened these to 17.0–229.8 and 19.7–177.3, respectively.

**Conclusion:**

Nationwide EAI dispensing provides the first all-age benchmark of access to community emergency epinephrine in Greece. By combining observed pack flow with transparent scenario-based analyses, this study introduces a reproducible approach for gaining insights into anaphylaxis epidemiology and emergency-treatment preparedness from national dispensing data, particularly where comprehensive registries are unavailable.

## Introduction

Anaphylaxis is a rapid-onset, potentially fatal systemic hypersensitivity reaction for which intramuscular epinephrine is first-line treatment and prompt community administration is central to preventing avoidable morbidity and mortality. International and European guidance emphasizes that patients at risk should have access to epinephrine auto-injectors (EAIs), be trained in their correct use, and receive appropriate follow-up after an anaphylaxis event (1–3). For clinicians and health systems, therefore, EAI availability is not merely a prescribing outcome. It is a practical marker of whether patients at risk are equipped for emergency self-management outside health-care facilities.

Despite this clinical importance, population-level surveillance of anaphylaxis remains difficult. Emergency department series, hospital discharge datasets, allergy registries, and insurance claims each capture only part of the burden, and their estimates vary according to case definition, coding practice, age group, setting, and health-care-seeking behavior (4). Comprehensive diagnosis-linked anaphylaxis registries are unavailable in many countries. This creates a recurrent problem for clinicians, allergy services, and health systems: national preparedness must be planned for a condition whose true burden is incompletely measured.

EAI dispensing data offer a complementary approach because they capture a clinically relevant downstream endpoint in allergy care. Simons and colleagues showed more than two decades ago that epinephrine dispensing data could provide a practical community-based approach to studying and monitoring the epidemiology of anaphylaxis (5). Subsequent work has used prescribing, dispensing, or acquisition data to describe EAI use, access, and regional or temporal trends in Australia, Sweden, Japan, Portugal, the United Kingdom, the United States, and Italy (6–12). These studies demonstrate that medication-use data can reveal system-level patterns not visible in clinical case series alone, including changes in access, age- or strength-specific utilization, prescriber behavior, regional variation, and the consequences of reimbursement or policy changes.

However, dispensing data must be interpreted cautiously. EAI dispensing is not equivalent to a confirmed diagnosis of anaphylaxis, and a dispensed pack does not represent a single patient or event. Pack flow may include post-reaction prescribing, precautionary prescribing for persons at risk, duplicate dispensing for home, school, or work, caregiver-held devices, expiry replacement, and stock reversals. Conversely, some clinically recognized anaphylaxis episodes are not followed by EAI acquisition or allergy follow-up (13–17). Thus, national pack flow cannot determine the appropriateness of prescribing for individual patients. Its value lies in distinguishing directly observed medication-use outcomes from assumption-based interpretations and in identifying whether a health system is generating a measurable signal of emergency treatment access for people at risk of anaphylaxis.

Greece has no comprehensive population-based estimate of anaphylaxis incidence or prevalence. Existing Greek evidence is important but partial, arising mainly from pediatric department surveys and registry-based pediatric and adolescent data rather than complete national surveillance (18, 19). During 2015–2017, a single EAI product was marketed nationally under emergency-import procedures, creating an unusual opportunity to examine nationwide EAI pack flow. The objective of this study was to quantify national EAI dispensing as a drug-utilization and emergency-treatment-access benchmark and, under explicit assumptions, to derive an incidence-compatible anaphylaxis indicator and a prevalence-equivalent active EAI coverage indicator. The study was designed to provide epidemiologically relevant insights into anaphylaxis in Greece rather than direct estimates of incidence or prevalence. Observed national net pack flow was primary, secondary analyses comprised population-level utilization by EAI strength and a full-labeled-shelf-life expiry-timing scenario, and scenario mapping was exploratory and hypothesis-generating.

## Materials and methods

### Study design and data source

A nationwide retrospective drug-utilization study was conducted using Anapen® DUO dispensing data in Greece from March 2015 through March 2017. The unit of observation was the monthly national net pack count. The dataset combined reimbursed dispensing claims and non-reimbursed sales and included retail and hospital pharmacy channels nationwide. During the study period, Anapen® supply was under emergency-import procedures, and the National Organization for the Provision of Health Services (EOPYY) issued prescribing instructions for emergency-import packs (20). EOPYY is Greece's major statutory health insurer, covering more than 95% of the population (21). Anapen® DUO was the only EAI product marketed in Greece during the observation window.

### Data sources, completeness, quality control, and reproducibility

The aggregate source tables were supplied by ALLERTEC HELLAS S.A. and comprised: i) a national monthly summary for March 2015-March 2016, ii) 12 monthly provider reports for April 2016-March 2017, and iii) an annual regional aggregation for April 2016-March 2017. The files reported net pack counts after returns and stock reversals and covered 25 consecutive months.

Quality-control reconciliation was performed before analysis. Monthly counts were summed by strength and period; the March 2015-March 2016 totals were reconciled with the first-year national summary; the April 2016-March 2017 national totals were reconciled with the annual regional aggregation; and period-specific sums were reconciled with the supplied 25-month strength-specific totals. Negative monthly values, representing months in which returns exceeded positive pack flow, were retained as supplied. No imputation or smoothing was applied. The author independently performed all derivations and analyses. Although the underlying commercial source tables cannot be publicly deposited, the reported summary counts, population denominators, equations, parameter values, and sensitivity ranges allow reproduction of the principal rates and scenario outputs.

### Analytic hierarchy and directly observed outcomes

To distinguish observation from interpretation, outcomes were organized into three tiers. Tier 1 comprised directly observed or directly derived drug-utilization outcomes. Tier 2 comprised population-level utilization rates by EAI strength and a full-labeled-shelf-life replacement-timing scenario. Tier 3 comprised exploratory deterministic scenario sensitivity analyses. Each Anapen® DUO pack contains two devices. The primary Tier 1 outcomes were: i) cumulative net pack counts over the 25-month observation window, ii) the annualized national net dispensing rate in packs per 100,000 person-years, and iii) the end-of-study 12-month net dispensing rate for April 2016-March 2017.

### Population-level utilization analysis by EAI strength

Because the dispensing records were not linked to individual recipients, utilization by EAI strength was analyzed only at the population level (ecologically). Counts of 150- and 300-microgram packs were related to age-equivalent population denominators derived from a prespecified 25 kg transition threshold within the 25-30 kg range recognized by guidance and product information (22–25). The threshold was translated into approximate age by linear interpolation between sex-specific median weight-for-age values from a published Greek growth reference, and the resulting estimates were cross-checked against World Health Organization and Centers for Disease Control and Prevention growth references (26–29) (Supplementary Appendix 1 and Supplementary Table S1).

### Expiry-replacement timing scenario

To examine how much recorded flow could arise from within-window expiry replacement, a timing scenario assumed that each pack had its full labeled shelf life available at dispensing: 21 months for Anapen® Junior 150 micrograms and 24 months for Anapen® 300 micrograms (24, 25). Packs dispensed early enough to reach label expiry before follow-up ended were counted, and each was hypothetically replaced once. Because residual shelf life at dispensing was unavailable, this calculation is not an estimate or absolute upper bound on actual expiry-driven replacement; it is a transparent scenario under the full-shelf-life assumption. Strength-specific counts are reported in Supplementary Table S2.

### Exploratory scenario sensitivity analyses

The scenario analysis was not a fitted epidemiologic model: no parameter was estimated from the aggregate Greek pack counts, and every output was a deterministic value conditional on stated assumptions. It translated the observed annualized pack rate into: i) an incidence-compatible anaphylaxis indicator and ii) a prevalence-equivalent active EAI coverage indicator. Parameter ranges were selected to make uncertainty auditable rather than to claim national calibration. The incident pathway-capture reference range (cI, 0.55-0.75) bracketed the 66.9% EAI prescription rate reported after pediatric anaphylaxis in Greece (18). Because prescription does not ensure acquisition, the 46.5% dispensing rate after United States emergency-department anaphylaxis visits (16) informed the broader cI sensitivity range of 0.40-0.90. The ranges for packs per captured episode (k, 1.0-1.5) and packs per covered person (m, 1.0-2.0) reflected one two-device twin-pack as the base unit and possible duplicate location- or caregiver-based dispensing (13, 14).

The strict-indication attribution parameters (sI and sE, 0.35-0.45) were not identifiable from the aggregate dataset and were therefore treated only as illustrative reference bands, not Greek estimates. The same numerical envelope was applied separately to incident pack flow and active pack stock for transparency; it does not imply that sI and sE are equal. In addition to combined reference-range analysis, one-at-a-time sensitivity analyses and deliberately broader assumption ranges were examined using sI/sE of 0.20-0.60, k of 1.0-2.0, cI of 0.40-0.90, and m of 1.0-3.0. The broader ranges show how conditional outputs change when uncertain assumptions are relaxed; they are not a second set of estimates. Full definitions, equations, rationale, calibration checks, and numerical results are provided in Supplementary Appendix 2 and Supplementary Tables S3-S4. Scenario ranges are not confidence intervals and should not be interpreted as national incidence or prevalence estimates.

### Population denominators and statistical analysis

National population denominators were derived from Hellenic Statistical Authority resident population counts from the 2011 and 2021 censuses. Linear intercensal interpolation gave a study-period denominator of 10,648,355 residents (30, 31). Detailed denominator and rate calculations are provided in Supplementary Appendix 3. Analyses were descriptive. The supplied records were treated as an enumeration of national pack flow across the described channels rather than as a probability sample, and net counts incorporated returns; therefore, sampling-based confidence intervals were not calculated. The rate ratio comparing the two EAI strengths was calculated descriptively from the two population-level, denominator-mapped rates. All calculations and figures were produced in R (R Foundation for Statistical Computing, Vienna, Austria). Reporting followed the Strengthening the Reporting of Observational Studies in Epidemiology (STROBE) statement (32).

### Ethics

The study used fully aggregated pack-count data without individual-level identifiers and involved no human-subject recruitment or direct patient contact. Under the institutional policy of 424 General Military Training Hospital, analysis of non-identifiable aggregate medication-flow data of this type does not require formal ethics committee review; therefore, no approval number was issued and informed consent was not applicable.

## Results

### Observed national pack flow

Over 25 months, 33,986 Anapen® DUO twin-packs were recorded nationally: 24,318 300-microgram packs and 9,668 150-microgram packs. The annualized national net dispensing rate was 153.2 packs per 100,000 person-years. During the final 12 months, 16,397 packs were recorded, corresponding to 154.0 packs per 100,000 population. Monthly net counts are shown in Figure 1, and the principal outcomes are summarized in Table 1.

**Figure 1:**
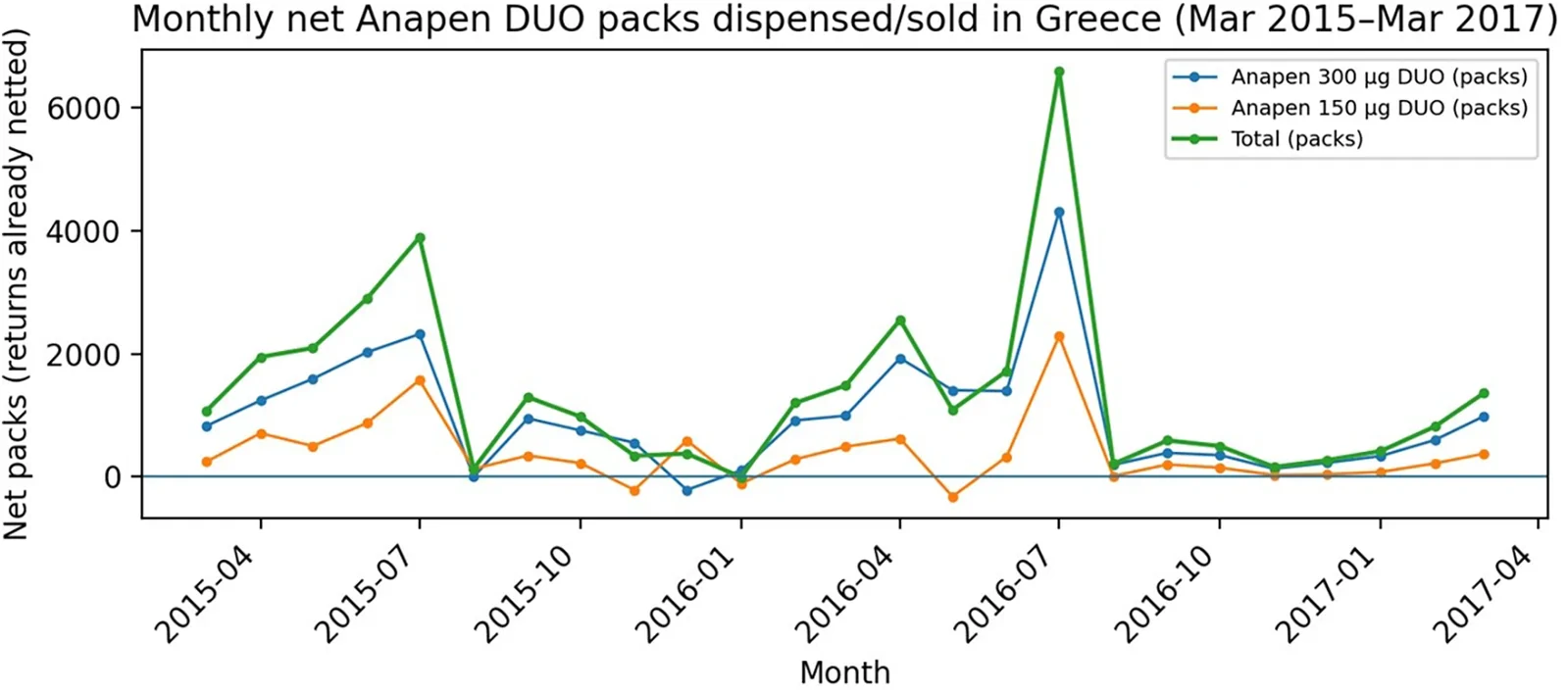
Monthly net Anapen® DUO pack counts in Greece (March 2015-March 2017). Lines show monthly net pack counts by strength and total. Negative values reflect months in which returns exceeded positive pack flow under net-of-returns accounting.

**Table 1:** National EAI drug-utilization outcomes, population-level analyses, and exploratory indicators relevant to anaphylaxis epidemiology and preparedness.

Indicator	Value
**Directly observed or directly derived national pack-flow outcomes**	
Observation window	March 2015-March 2017 (25 months)
Total net packs	33,986
300-microgram net packs	24,318
150-microgram net packs	9,668
Annualized national net dispensing rate	153.2 packs per 100,000 person-years
End-of-study 12-month net dispensing rate	154.0 packs per 100,000 population
Secondary full-labeled-shelf-life replacement-timing scenario	
Packs eligible for one within-window replacement under timing scenario	3,156 packs (9.3% of total pack flow)
**Population-level utilization by EAI strength**	
Primary strength-denominator mapping	25 kg approximately 6.1-6.3 completed years; operationalized as younger than 7 years versus 7 years or older
150-microgram age-equivalent denominator	680,845 persons younger than 7 years
300-microgram age-equivalent denominator	9,967,510 persons aged 7 years or older
Annualized 150-microgram denominator-mapped utilization rate	681.6 packs per 100,000 person-years
Annualized 300-microgram denominator-mapped utilization rate	117.1 packs per 100,000 person-years
Rate ratio between denominator-mapped strength rates	5.8-fold higher 150-microgram than 300-microgram denominator-mapped rate
Exploratory scenario-derived epidemiologic indicators	
Incidence-compatible anaphylaxis indicator, reference ranges	47.7–125.3 episodes per 100,000 person-years
Prevalence-equivalent active EAI coverage, reference ranges	51.7–133.0 persons per 100,000 population (0.052–0.133%)
Incidence-compatible anaphylaxis indicator, broader assumption ranges	17.0–229.8 episodes per 100,000 person-years
Prevalence-equivalent active EAI coverage, broader assumption ranges	19.7–177.3 persons per 100,000 population

EAI, epinephrine auto-injector.

Details: population-level mapping by EAI strength, Supplementary Appendix 1 and Supplementary Table S1; expiry timing scenario, Supplementary Table S2; scenario equations, calibration, and sensitivity analyses, Supplementary Appendix 2 and Supplementary Tables S3-S4; denominator calculations, Supplementary Appendix 3.

### Population-level utilization by EAI strength

In the Greek growth reference, the prespecified 25 kg threshold corresponded to approximately 6.1 completed years for boys and 6.3 completed years for girls. Because both values fell within the completed 6-year-old band, the analysis operationalized 150-microgram packs against the population younger than 7 years (680,845 persons) and 300-microgram packs against the population aged 7 years or older (9,967,510 persons) (Supplementary Appendix 1 and Supplementary Table S1).

Using the age-equivalent population denominators, the annualized utilization rates were 681.6 packs per 100,000 person-years for the 150-microgram strength and 117.1 packs per 100,000 person-years for the 300-microgram strength, yielding a 5.8-fold ratio between the two denominator-mapped rates.

### Expiry-replacement timing scenario

Under the full-labeled-shelf-life timing scenario, 3,156 packs were dispensed early enough to reach label expiry within the 25-month observation window. If each were replaced once, the corresponding replacement volume would equal 9.3% of total recorded pack flow (Supplementary Table S2).

### Exploratory scenario sensitivity outputs

Across all combinations of the reference ranges, the incidence-compatible anaphylaxis indicator was 47.7-125.3 episodes per 100,000 person-years, and prevalence-equivalent active EAI coverage was 51.7-133.0 persons per 100,000 population (0.052%-0.133%). In practical terms, the first indicator asks how many annual anaphylaxis episodes would be mathematically consistent with the observed pack flow after allowing for incomplete EAI acquisition and multiple packs, whereas the second asks how many persons could hold in-date packs after allowing for duplicate pack holdings. When wider ranges were used for strict-indication attribution, EAI acquisition, and the number of packs per episode or covered person, the corresponding outputs ranged from 17.0 to 229.8 episodes per 100,000 person-years and from 19.7 to 177.3 persons per 100,000 population. These wider ranges show how strongly the results depend on the assumptions used and should not be interpreted as a second set of estimates. Scenario equations, one-at-a-time analyses, broader assumption-range analyses, and calibration checks are reported in Supplementary Appendix 2 and Supplementary Tables S3-S4. Regional pack counts for the final 12 months are provided in Supplementary Figure S1.

## Discussion

This study provides the first nationwide, all-age epidemiologic window on anaphylaxis-related EAI use in Greece. It directly documents 153.2 Anapen® DUO twin-packs per 100,000 person-years after returns and stock reversals during 2015-2017, with a final 12-month rate of 154.0 packs per 100,000 population. Because EAIs are supplied to people with previous anaphylaxis or a clinically judged risk of future anaphylaxis, national pack flow reflects the health system's response to the population considered eligible for community emergency epinephrine. Packs are not unique persons or confirmed episodes, but the endpoint is nevertheless an anaphylaxis-related drug-utilization and preparedness indicator rather than merely a pharmaceutical sales measure.

The exploratory scenario framework extends this observed benchmark while keeping assumption dependence visible. Under the reference ranges, the incidence-compatible anaphylaxis indicator was 47.7-125.3 episodes per 100,000 person-years. This overlaps the current global meta-analytic estimate of 46 cases per 100,000 population per year (95% confidence interval, 21-103) and the 50-112 episodes per 100,000 person-years range summarized in World Allergy Organization guidance (2, 4). The overlap supports the external plausibility of the reference scenario but does not serve as direct validation. The intentionally broader assumptions expanded the compatible range to 17.0-229.8, clearly illustrating that event-oriented translation relies heavily on indication attribution, episode-to-acquisition capture, and packs per episode. This sensitivity is valuable because it allows uncertainty to be checked and verified, rather than hiding false accuracy within a single estimate.

The prevalence-equivalent translation yielded active EAI coverage of 51.7-133.0 persons per 100,000 (0.052%-0.133%) under the reference assumptions. This represents potential holders of in-date packs within the specific strict-indication framework, and does not include every individual eligible for an EAI. It is therefore expected to be lower than published lifetime anaphylaxis prevalence estimates, including 0.3%-5.1% in World Allergy Organization guidance, approximately 0.3% in a European systematic review, and 1.6%-5.1% in broader practice-parameter estimates (2, 3, 33). Related medication-use endpoints provide scale: 0.95% of a defined Canadian population had injectable epinephrine dispensed over 5 years (5), Australian annual rates of individuals with dispensed EAIs increased from 71.5 to 82.1 per 100,000 person-years (6), and a Portuguese acquisition study reported an anaphylaxis-occurrence proxy of 0.165% (9). The Greek construct adds a population-scaled benchmark of potential in-date emergency-treatment coverage that future diagnosis-linked research can test.

The contribution is clinical, epidemiologic, and methodological. Clinically, the study supplies a national benchmark for EAI distribution and emergency-treatment access. Epidemiologically, it provides an all-age population signal in a country without comprehensive diagnosis-linked surveillance. Methodologically, it separates observed pack flow from population-level analyses of aggregate data and explicitly assumption-conditioned translations. To our knowledge, no previous national study has derived both an incidence-compatible anaphylaxis indicator and a prevalence-equivalent active EAI coverage indicator from the same pack-flow series while reporting these as distinct from directly observed utilization. These indicators have not yet been validated against patient-level clinical data. However, the framework can be tested in future studies linking dispensing records to diagnoses and patient outcomes.

Population medication-flow data are an established complement to case-based surveillance. Simons and colleagues described epinephrine dispensing as a practical community approach to studying and monitoring the epidemiology of anaphylaxis (5), and subsequent national or population-based studies have examined access, temporal trends, age patterns, and policy effects (6–12). A key advantage is that EAI dispensing captures a downstream clinical action across the community, including both survivors of prior anaphylaxis and individuals judged to have a clinically meaningful future risk, rather than only events reaching a particular hospital or registry (1–3, 13–15).

The Greek data source strengthens national interpretability. During the study interval, Anapen® DUO was the only EAI marketed nationally, the records covered reimbursed and non-reimbursed retail and hospital channels, and EOPYY covers more than 95% of the population (20, 21). These features reduce fragmentation and allow the captured pack flow to serve as an unusually broad national distribution benchmark. They do not demonstrate that every prescribed pack was acquired, held, or used by the intended patient, but they support the epidemiologic relevance of the medication-use signal. The endpoint measures whether emergency epinephrine enters the community. It does not measure correct patient selection, carriage, training, recognition of anaphylaxis, or timely administration.

Because EAI dose selection is weight-guided, relating 150- and 300-microgram pack flow to age-equivalent denominators provides a population-level comparison between denominator-mapped lower-weight/younger and higher-weight/older population strata. The 5.8-fold contrast therefore demonstrates substantially greater 150-microgram dispensing intensity relative to its mapped population denominator, although no recipient-level age or weight linkage was available. Age-related EAI dispensing patterns have also been reported in Australia, the United States, and the United Kingdom (6, 10, 11, 16), and children younger than 5 years were more likely to have an EAI dispensed after an emergency-department anaphylaxis visit in United States claims data (16). Higher food-allergy burden in young children, caregiver risk perception, school requirements, specialist contact, and multi-location supply are plausible contributors (14, 34–36), but no direct evidence establishes that parents acquire EAIs more reliably for children than adults do for themselves. Recent English prescription research used age as a proxy for weight to examine dose appropriateness (37), supporting the analytical principle. The present study extends it by constructing nationwide population denominators for dispensing by EAI strength. This aggregate comparison is therefore hypothesis-generating and cannot establish age-specific anaphylaxis incidence, prescribing appropriateness, differential preparedness, or the mechanism responsible for the difference.

For Greek allergy care, the observed rates establish a baseline that was previously unavailable. Existing Greek studies described pediatric anaphylaxis management and registry cases but not a nationwide medication-access signal across age groups (18, 19). Future work can test whether patients discharged after anaphylaxis obtain EAIs and allergy follow-up, whether devices are renewed and carried, how prescribing aligns with age, weight, diagnosis, and guideline-based risk, and whether regional supply differences reflect access barriers or reporting practices. The present aggregate data cannot answer those patient-level questions, but they identify the variables that clinically linked surveillance should capture.

The framework may also be useful in other countries lacking comprehensive registries, provided that product availability and channel coverage are documented. Pharmacy, reimbursement, procurement, and sales datasets can supply an initial epidemiologic and preparedness benchmark. Their interpretability would improve substantially through linkage to age, indication, prescriber specialty, emergency-department encounters, hospital diagnoses, allergy follow-up, and renewals, allowing aggregate medication-use proxies to evolve into patient-level measures of post-anaphylaxis care quality.

Several design features strengthen interpretation: nationwide scope during a single-product interval, inclusion of multiple reimbursed and non-reimbursed channels, net-of-returns accounting, internal reconciliation across source summaries, and explicit reporting of equations and sensitivity ranges. These features support the directly observed national pack-flow benchmark. The principal interpretive limit is the aggregate nature of the data: counts could not be linked to diagnoses, indications, episodes, prescribers, age, body weight, unique patients, adherence, training, carriage, or actual use. A pack may represent an initial acquisition, precautionary prescribing, duplicate location or caregiver supply, renewal, or replacement; conversely, a recognized episode may not lead to EAI acquisition. Some anaphylaxis episodes may also remain unrecognized when the presentation is attributed to another acute diagnosis or is managed outside allergy services, further weakening any one-to-one relationship between event burden and subsequent dispensing. These constraints preclude patient-level and causal inference, but they do not invalidate the observed enumeration of net pack flow within the described channels.

Capture was broad but lacked independent auditing against patient-level claims or clinical records. EOPYY coverage of more than 95% of the population, inclusion of retail and hospital channels, and the single marketed product mitigate fragmentation without proving complete capture. Emergency-import availability, reimbursement, patient cost, prescriber behavior, access to pharmacies and allergy services, shipment timing, and returns could affect pack flow. The mid-2016 peak and occasional negative monthly values confirm that the endpoint is net distribution intensity rather than patient acquisition. Residual shelf life at dispensing was unknown. Consequently, the expiry-replacement calculation is a full-labeled-shelf-life timing scenario rather than an absolute upper bound on actual replacement. Regional categories were distributor-defined and regional population denominators were unavailable. These factors limit interpretation of clinical demand but do not alter the arithmetic national net-flow benchmark.

The population-level and scenario analyses are novel but conditional extensions. The 25 kg mapping is a practical convention within a 25-30 kg transition band and supports an aggregate contrast, but it cannot classify individual recipients. The strict-indication attribution bands were illustrative rather than estimated from Greek data, and deliberately broader assumption ranges produced markedly wider outputs. Sampling-based confidence intervals were not calculated because the supplied records were treated as an enumeration of the described channels rather than a probability sample. Structural uncertainty is instead displayed through transparent sensitivity ranges. Accordingly, the scenario outputs should not be used as point estimates of national disease burden, but they remain useful for hypothesis generation, external comparison, and the design of linked surveillance.

Finally, dispensing volume captures only one component of preparedness. Comprehensive preparedness requires accurate risk assessment, appropriate prescribing, patient and caregiver education, device availability and carriage, recognition of anaphylaxis, rapid epinephrine administration, emergency response, and specialist follow-up. Nevertheless, whether emergency epinephrine enters the community is a necessary and measurable component. Because this endpoint is clearly defined and can be measured consistently at the population level, it provides a useful public-health baseline for developing more clinically linked surveillance.

## Conclusion

Nationwide EAI pack flow offers, to our knowledge, the first comprehensive countrywide benchmark of community emergency-epinephrine access for all ages in Greece. By separating actual dispensing data from population-level analyses and assumption-based conversions, the study presents a reproducible dual framework. This framework helps derive an incidence-compatible anaphylaxis indicator and a prevalence-equivalent active EAI coverage indicator, especially when diagnosis-linked surveillance is lacking. These indicators do not replace confirmed incidence or prevalence but provide clinically relevant insights into the epidemiology of anaphylaxis using national medication-use signals. The EAI strength mapping provides a testable population-level finding, while the framework also highlights patient-level linkages necessary for future validation.

## Data Availability

The data analyzed in this study is subject to the following licenses/restrictions: The aggregated source data were provided by a third party and are not publicly posted. Derived summary data sufficient to reproduce the reported calculations are contained within the article and the Supplementary Material. Additional derived tabulations and analytic code are available from the corresponding author upon reasonable request, subject to the data provider's permission. Requests to access these datasets should be directed to gnkonstantinou@gmail.com.
