# Supplementary material for: Nationwide epinephrine auto-injector dispensing in Greece: a drug-utilization study providing insights into anaphylaxis epidemiology and emergency-treatment preparedness
Source: Front Allergy. 2026 Sep 14;7:1906574. doi: 10.3389/falgy.2026.1906574 (PMC13616777; doi:10.3389/falgy.2026.1906574)
Supplement: Supplementary file 1 [file Supplementaryfile1.docx]

# SUPPLEMENTARY MATERIAL

## Supplementary Appendix 1

### Population-level mapping of EAI strength to an approximate age denominator

Because the dataset does not include age or weight at the individual level, EAI strength was used only to construct a population-level utilization comparison (i.e., an ecological analysis based on aggregate pack counts and denominators). For the primary denominator mapping, the lower transition threshold of 25 kg was prespecified, consistent with guidance and product information supporting adult-dose transition within the 25-30 kg band (22-25). Age-equivalent denominators were approximated using WHO, CDC, and Greek growth references (26-29).

In the Greek LMS reference, linear interpolation between the 6- and 7-year median weights placed 25 kg at approximately 6.1 completed years for boys and 6.3 completed years for girls. Because both values fell within the completed 6-year-old band, the primary analysis operationalized 150-microgram packs against the population younger than 7 years and 300-microgram packs against the population aged 7 years or older. This aggregate mapping does not establish the age or weight of any recipient and must not be used to infer individual prescribing appropriateness, age-specific disease burden, or differential preparedness.

**Supplementary Table S1.** Population-level utilization mapping by EAI strength using the 25 kg transition threshold

| Mapping element | Value used | Rationale/derivation |
| --- | --- | --- |
| Weight threshold | 25 kg | Lower bound of the 25-30 kg transition band used for primary mapping |
| Age equivalent, boys | Approximately 6.1 completed years | Linear interpolation between Greek LMS median weights: 24.71 kg at age 6 and 27.85 kg at age 7 |
| Age equivalent, girls | Approximately 6.3 completed years | Linear interpolation between Greek LMS median weights: 24.18 kg at age 6 and 27.30 kg at age 7 |
| Operational denominator for 150 micrograms | Younger than 7 years; 680,845 persons | Population below the age-equivalent threshold |
| Operational denominator for 300 micrograms | 7 years or older; 9,967,510 persons | Total annualized denominator minus the younger-than-7 denominator |
| Interpretation | Population-level utilization proxy by EAI strength | Not an individual age, weight, diagnosis, indication, or prescribing-appropriateness classification |

The expiry-timing analysis assumed that the full labeled shelf life remained available at dispensing. Packs issued early enough to reach label expiry before the end of follow-up were counted and each was hypothetically replaced once. Because residual shelf life at dispensing was unavailable, this is a full-labeled-shelf-life replacement-eligibility scenario, not an estimate or absolute upper bound on actual expiry-driven replacement.

**Supplementary Table S2.** Full-labeled-shelf-life replacement-eligibility scenario by strength

| Strength | Observed packs | Packs eligible under timing scenario | Share of total pack flow |
| --- | --- | --- | --- |
| 150 micrograms | 9,668 | 1,103 | 3.2% |
| 300 micrograms | 24,318 | 2,053 | 6.0% |
| Total | 33,986 | 3,156 | 9.3% |

## Supplementary Appendix 2

### Exploratory deterministic scenario framework for deriving indicators relevant to anaphylaxis epidemiology and preparedness from EAI dispensing

Exploratory deterministic scenario analyses translated the observed annualized national EAI pack rate into two conditional indicators: an incidence-compatible anaphylaxis indicator and a prevalence-equivalent active EAI coverage indicator. Each indicator was calculated directly from prespecified parameter values representing strict-indication attribution, packs per captured episode or covered person, episode-to-acquisition capture, and active-pack duration.

The framework comprised three complementary components: combined analysis across all reference-range combinations, one-at-a-time sensitivity analyses around reference midpoints, and a broader assumption-range sensitivity analysis. Parameter values were specified independently of the aggregate Greek pack counts and were informed by the Anapen DUO pack configuration, clinical-practice considerations, Greek and international evidence on post-anaphylaxis EAI prescribing or acquisition, and deliberately broad bounds for unmeasured attribution parameters. The resulting ranges characterize the order of magnitude compatible with the observed pack flow under each stated parameter set.

### Definitions

**D:** Observed annualized national net EAI pack rate (packs per 100,000 person-years); D = 153.2.

**D12:** End-of-study 12-month net EAI pack rate (packs per 100,000 population); D12 = 154.0.

**L:** Volume-weighted labeled shelf life. The analysis used 24 months for 300-microgram packs and 21 months for 150-microgram packs, giving L = 1.9289 years (approximately 1.93) (24,25).

**H:** Steady-state proxy for active packs in the community (packs per 100,000), approximated as H = D x L under a stationarity assumption.

**sI:** Fraction of annual pack flow attributed, within a scenario, to incident episodes followed by a strict EAI indication rather than precautionary prescribing, duplicate dispensing, or replacement.

**k:** Packs attributed to each captured incident anaphylaxis episode along the episode-to-acquisition pathway.

**cI:** Probability that a clinically recognized incident episode leads to acquisition of at least one EAI pack.

**sE:** Fraction of active pack stock attributed, within a scenario, to persons covered under a strict EAI-indication construct.

**m:** Packs per covered person under the strict EAI-indication construct.

### Reference parameter selection and rationale

Strict-indication attribution (sI and sE), 0.35-0.45. These parameters were not identifiable from the aggregate dataset and were specified only as illustrative reference bands representing a minority-to-substantial-minority share of annual pack flow or active pack stock within the strict-indication construct. The same numerical envelope was applied separately to sI and sE for transparency and does not imply that the incident-flow and active-stock attribution fractions are equal. The broader assumption-range analysis evaluates values from 0.20 to 0.60.

Packs per captured incident episode (k), 1.0-1.5. Each Anapen DUO pack contains two devices, making one twin-pack the base acquisition unit after a captured episode. Values above 1 represent additional twin-packs for separate households, schools, workplaces, caregivers, or other locations, consistent with clinical guidance and discussion of who requires EAIs (13,14).

Incident pathway capture (cI), 0.55-0.75. The reference range brackets the 66.9% EAI prescription rate reported after pediatric anaphylaxis in a Greek nationwide survey (18). Because prescription does not ensure acquisition, United States claims data documenting EAI dispensing after 46.5% of emergency-department anaphylaxis visits (16) informed the broader cI sensitivity range of 0.40-0.90 rather than the reference band. These studies are contextual anchors across different ages, settings, indications, and health systems; neither is treated as a calibrated Greek acquisition estimate.

Packs per covered person (m), 1.0-2.0. The range spans one two-device twin-pack per covered person to duplicate twin-packs held across locations or caregivers, reflecting practical prescribing arrangements described in clinical guidance (13,14).

### Broader assumption-range sensitivity analysis

Broader ranges were evaluated to show structural sensitivity beyond the illustrative reference envelope: sI/sE = 0.20-0.60, k = 1.0-2.0, cI = 0.40-0.90, and m = 1.0-3.0. These deliberately expanded bounds show how the conditional outputs move when uncertain assumptions are relaxed; they do not constitute a second set of estimates.

### Computation

The incidence-compatible anaphylaxis indicator (episodes per 100,000 person-years) was calculated as I = D x sI / (k x cI).

The prevalence-equivalent active EAI coverage indicator (persons per 100,000 population) was calculated as E = (D x L) x sE / m.

The prevalence-equivalent active EAI coverage indicator represents the number of persons per 100,000 population whose active-pack holdings are compatible with the strict-indication construct. The term prevalence-equivalent refers to its person-based population denominator; estimating all persons with an EAI indication, including those without active packs, would require an additional coverage parameter.

### Numerical results and interpretation

Using D = 153.2 and L = 1.9289 gives H = 295.5 active packs per 100,000 population. Across all combinations of the reference ranges (sI = 0.35-0.45, k = 1.0-1.5, cI = 0.55-0.75), I = 47.7-125.3 episodes per 100,000 person-years. Across sE = 0.35-0.45 and m = 1.0-2.0, E = 51.7-133.0 persons per 100,000 population (0.052-0.133%).

At reference midpoints (sI = 0.40, k = 1.25, cI = 0.65; sE = 0.40, m = 1.5), I = 75.4 episodes per 100,000 person-years and E = 78.8 persons per 100,000 population. One-at-a-time sensitivity analyses show that each parameter materially changes the output (Supplementary Table S4). Combining the broader assumption-range extremes yields I = 17.0-229.8 and E = 19.7-177.3 per 100,000, demonstrating the breadth of values compatible with alternative parameter sets.

For descriptive calibration, the attribution share needed to reproduce an external benchmark incidence I* is sI = I* x k x cI / D. Using D = 153.2, I* = 46-112 per 100,000 person-years, k = 1.0, and cI = 0.65 gives sI = 0.20-0.48. Across k = 1.0-1.5 and cI = 0.55-0.75, the required share is approximately 0.17-0.82. This calculation provides an internal consistency check against the external incidence benchmarks (2,4).

**Supplementary Table S3.** Reference scenario and calibration outputs

| Analysis | sI or sE | k or m | cI | Deterministic output |
| --- | --- | --- | --- | --- |
| Incidence-compatible anaphylaxis indicator | 0.35-0.45 | k = 1.0-1.5 | 0.55-0.75 | 47.7-125.3 episodes per 100,000 person-years |
| Prevalence-equivalent active EAI coverage indicator | 0.35-0.45 | m = 1.0-2.0 | Not used | 51.7-133.0 persons per 100,000 population (0.052-0.133%) |
| Reference calibration attribution share | Derived | k = 1.0 | 0.65 | 20-48% of pack flow |
| Broad calibration attribution share | Derived | k = 1.0-1.5 | 0.55-0.75 | 17-82% of pack flow |

**Supplementary Table S4.** One-at-a-time and broader assumption-range sensitivity analyses

| Output | Scenario varied | Other values held at reference midpoint | Deterministic result |
| --- | --- | --- | --- |
| Incidence-compatible indicator | Reference midpoint | sI = 0.40; k = 1.25; cI = 0.65 | 75.4 per 100,000 person-years |
| Incidence-compatible indicator | sI = 0.35-0.45 | k = 1.25; cI = 0.65 | 66.0-84.8 per 100,000 person-years |
| Incidence-compatible indicator | k = 1.0-1.5 | sI = 0.40; cI = 0.65 | 62.9-94.3 per 100,000 person-years |
| Incidence-compatible indicator | cI = 0.55-0.75 | sI = 0.40; k = 1.25 | 65.4-89.1 per 100,000 person-years |
| Incidence-compatible indicator | All reference extremes | Reference ranges combined | 47.7-125.3 per 100,000 person-years |
| Incidence-compatible indicator | Broader assumption ranges | sI = 0.20-0.60; k = 1.0-2.0; cI = 0.40-0.90 | 17.0-229.8 per 100,000 person-years |
| Prevalence-equivalent active EAI coverage indicator | Reference midpoint | sE = 0.40; m = 1.5 | 78.8 per 100,000 population |
| Prevalence-equivalent active EAI coverage indicator | sE = 0.35-0.45 | m = 1.5 | 69.0-88.7 per 100,000 population |
| Prevalence-equivalent active EAI coverage indicator | m = 1.0-2.0 | sE = 0.40 | 59.1-118.2 per 100,000 population |
| Prevalence-equivalent active EAI coverage indicator | All reference extremes | Reference ranges combined | 51.7-133.0 per 100,000 population |
| Prevalence-equivalent active EAI coverage indicator | Broader assumption ranges | sE = 0.20-0.60; m = 1.0-3.0 | 19.7-177.3 per 100,000 population |

All values in Tables S3-S4 are deterministic scenario outputs conditional on the specified assumptions. The reported ranges summarize variation across parameter sets and are intended to provide insights into anaphylaxis epidemiology and preparedness without being interpreted as confidence intervals or direct national incidence or prevalence estimates.

## Supplementary Appendix 3

### Population denominators and rate calculations

National population denominators were derived from Hellenic Statistical Authority resident population counts for the 2011 census (10,816,286) and the 2021 census (10,482,487) (30,31). Linear intercensal interpolation for the study window gave the population denominator used in the rate calculations: 10,648,355.

For the 25-month annualized analysis, person-years = 10,648,355 x 25/12. The annualized national rate = 33,986 / (10,648,355 x 25/12) x 100,000 = 153.2 packs per 100,000 person-years.

The end-of-study 12-month rate = 16,397 / 10,648,355 x 100,000 = 154.0 packs per 100,000 population.

## Supplementary figure caption

Supplementary Figure S1. Regional net Anapen DUO pack counts in Greece during the final 12 months (April 2016-March 2017), by strength. Regions follow distributor-defined reporting categories; no regional population denominators were available.
