# Supplementary material for: Nationwide epinephrine auto-injector dispensing in Greece: a drug-utilization study providing insights into anaphylaxis epidemiology and emergency-treatment preparedness
Source: Front Allergy. 2026 Sep 14;7:1906574. doi: 10.3389/falgy.2026.1906574 (PMC13616777; doi:10.3389/falgy.2026.1906574)
Supplement: Supplementary file 2 [file Supplementaryfile2.docx]

# STROBE Statement checklist for cohort, case-control, and cross-sectional studies

| **STROBE item** | **Item no.** | **Location in revised manuscript** |
| --- | --- | --- |
| Title and abstract | 1 | Title page and Abstract; study design and exploratory status stated explicitly |
| Background/rationale | 2 | Introduction |
| Objectives | 3 | Introduction, final paragraph |
| Study design | 4 | Materials and methods: Study design and data source; Analytic hierarchy and directly observed outcomes |
| Setting | 5 | Materials and methods: Study design and data source |
| Participants | 6 | Not applicable; aggregate national pack-count study without individual participants |
| Variables | 7 | Materials and methods: Analytic hierarchy; Population-level utilization analysis by EAI strength; Expiry-replacement timing scenario; Exploratory scenario sensitivity analyses |
| Data sources/measurement | 8 | Materials and methods: Data sources, completeness, quality control, and reproducibility; Supplementary Material |
| Bias | 9 | Discussion: expanded limitations, including clinical under-ascertainment, supply-chain effects, and model uncertainty |
| Study size | 10 | Materials and methods and Results; all supplied national pack-count records for 25 consecutive months were included |
| Quantitative variables | 11 | Materials and methods: Population-level utilization analysis by EAI strength; Exploratory scenario sensitivity analyses |
| Statistical methods | 12 | Materials and methods: Population denominators and statistical analysis (descriptive rates; no sampling-based confidence intervals); Supplementary Appendices 2-3 and Tables S3-S4 |
| Participants/descriptive data/outcome data | 13-16 | Results; Table 1; Figure 1; Supplementary Figure S1 |
| Main results | 17 | Results; Table 1 |
| Other analyses | 18 | Results: Exploratory scenario sensitivity outputs; Supplementary Appendix 2 and Tables S3-S4 |
| Key results | 19 | Discussion, opening paragraph |
| Limitations | 20 | Discussion, expanded strengths and balanced limitations section |
| Interpretation | 21 | Discussion; explicit separation of observed, population-level, and scenario findings |
| Generalisability | 22 | Discussion and Conclusion |
| Funding | 23 | Funding statement |
